# Characterization of Bean Necrotic Mosaic Virus: A Member of a Novel Evolutionary Lineage within the Genus *Tospovirus*

**DOI:** 10.1371/journal.pone.0038634

**Published:** 2012-06-08

**Authors:** Athos Silva de Oliveira, Fernando Lucas Melo, Alice Kazuko Inoue-Nagata, Tatsuya Nagata, Elliot Watanabe Kitajima, Renato Oliveira Resende

**Affiliations:** 1 Department of Cell Biology, University of Brasília, Brasília, Distrito Federal, Brazil; 2 Embrapa Vegetables, Brasília, Distrito Federal, Brazil; 3 University of São Paulo, Piracicaba, São Paulo, Brazil; University of Kansas Medical Center, United States of America

## Abstract

**Background:**

Tospoviruses (Genus *Tospovirus*, Family *Bunyaviridae*) are phytopathogens responsible for significant worldwide crop losses. They have a tripartite negative and ambisense RNA genome segments, termed S (Small), M (Medium) and L (Large) RNA. The vector-transmission is mediated by thrips in a circulative-propagative manner. For new tospovirus species acceptance, several analyses are needed, e.g., the determination of the viral protein sequences for enlightenment of their evolutionary history.

**Methodology/Principal Findings:**

Biological (host range and symptomatology), serological, and molecular (S and M RNA sequencing and evolutionary studies) experiments were performed to characterize and differentiate a new tospovirus species, Bean necrotic mosaic virus (BeNMV), which naturally infects common beans in Brazil. Based upon the results, BeNMV can be classified as a novel species and, together with Soybean vein necrosis-associated virus (SVNaV), they represent members of a new evolutionary lineage within the genus *Tospovirus*.

**Conclusion/Significances:**

Taken together, these evidences suggest that two divergent lineages of tospoviruses are circulating in the American continent and, based on the main clades diversity (American and Eurasian lineages), new tospovirus species related to the BeNMV-SVNaV clade remain to be discovered. This possible greater diversity of tospoviruses may be reflected in a higher number of crops as natural hosts, increasing the economic impact on agriculture. This idea also is supported since BeNMV and SVNaV were discovered naturally infecting atypical hosts (common bean and soybean, respectively), indicating, in this case, a preference for leguminous species. Further studies, for instance a survey focusing on crops, specifically of leguminous plants, may reveal a greater tospovirus diversity not only in the Americas (where both viruses were reported), but throughout the world.

## Introduction

*Tospovirus* is the only plant-infecting genus of the family *Bunyaviridae* and its members are responsible for significant quality and yielding losses to crops worldwide [1]. The tospoviruses have enveloped quasi-spherical particles and a tripartite negative and ambisense RNA genome containing five open reading frames [2]. The genomic segments are denominated according to their size, as S (Small), M (Medium) and L (Large). The S RNA encodes a non-structural RNA-silencing suppressor protein (NSs) and the nucleocapsid protein (N) [3], [4]. The M RNA encodes a cell-to-cell movement protein (NSm) and the envelope glycoproteins precursor (GPp) [5], [6]. The L RNA encodes an RNA-dependent RNA polymerase (RdRp), also called L protein [7].

The tospoviruses are transmitted by thrips insects (Order Thysanoptera) in a circulative-propagative manner [8], [9]. Despite the existence of more than 5,000 thrips species, only 14 species are known as potential tospovirus vectors and most of them belong to the genera *Frankliniella* and *Thrips* [10]. The *Frankliniella* genus is neotropical with all but seven species considered endemic to the New World [11], while the worldwide distributed genus *Thrips* has no species native to South America [12]. Interestingly, the natural distribution of these vector species is somewhere reflected in the tospovirus phylogenetic relationships, with *Fankliniella*-transmitted tospoviruses clustering in an "American lineage"; and *Thrips-*transmitted tospoviruses clustering in an "Eurasian lineage"; [1]. Another evolutionary lineage is formed by two tospoviruses isolated from peanut and transmitted by thrips from genus *Scirtothrips* [13].

Recently, two new tospoviruses were described infecting soybean (*Glycine max* (L.) Merr.) [14] and common bean (*Phaseolus vulgaris* L.) [15]; both of them clustered together and apart from the other tospovirus lineages. While the soybean-infecting tospovirus (Soybean vein necrosis-associated virus, SVNaV) genome has been completely sequenced [14], only the RNA-dependent RNA polymerase gene of the common bean tospovirus (Bean necrotic mosaic virus, BeNMV) has been determined [15]. No biological characterization has been reported for either virus. Therefore, we carried out an extensive analysis of a BeNMV isolate, including the study of its host range, symptomatology, serological differentiation, and genome sequencing, revealing that, indeed, this virus is a representative of a new evolutionary lineage within genus *Tospovirus*.

## Results and Discussion

### Polyclonal antibodies against the nucleoprotein discriminate BeNMV from other Brazilian tospoviruses

**Figure 1:**
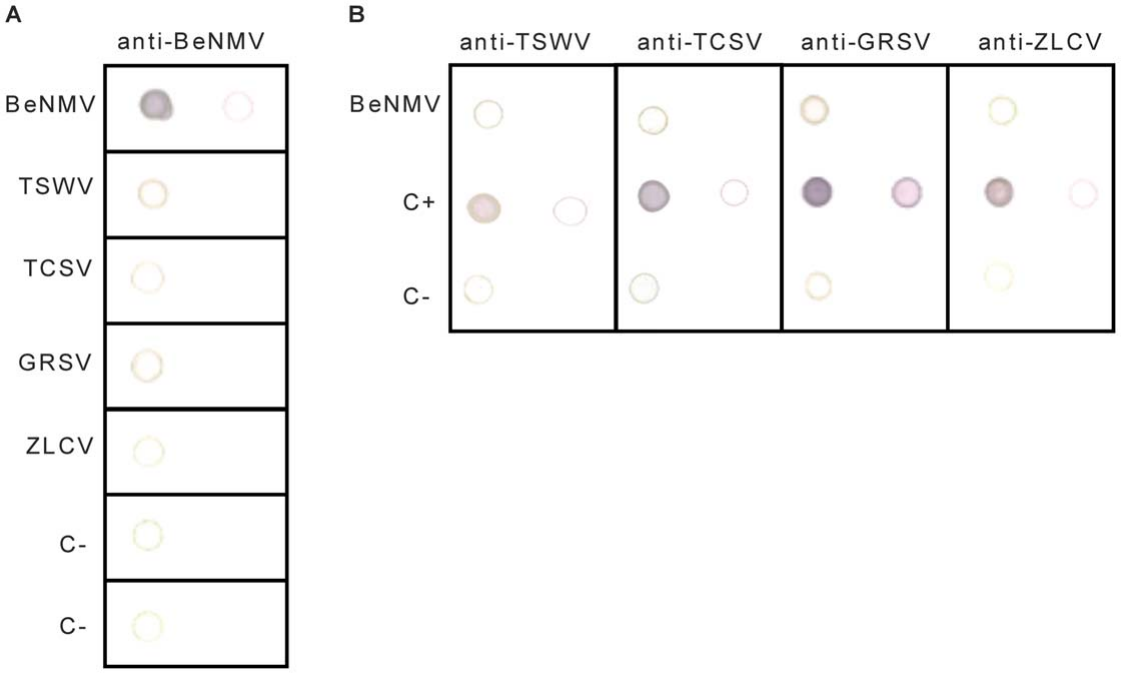
Serological differentiation between BeNMV and other Brazilian tospovirus species. A) Tospovirus-infected plant extract dots incubated with anti-BeNMV. The plant hosts were *Physalis pubescens* to BeNMV and *Datura stramonium* to the others. The negative controls (C−) are healthy *P. pubescens* and *D. stramonium*, respectively. B) Confirmation of the virus presence in the samples utilized and cross-reaction evaluation. The dots in the first column are 1∶100 dilutions (leaf mass per volume of 0.5× PBS (g/ml), while the second column are 1∶1000 dilutions.

After nucleocapsid (N) purification from BeNMV-infected *Physalis pubescens* L., a protein of approximately 29 kDa was visualized by sodium dodecyl sulfate polyacrylamide gel electrophoresis (SDS-PAGE) (data not shown). Polyclonal antibodies against this protein were produced in rabbits. The serological differentiation performed through dot enzyme linked immunosorbent assay (DOT-ELISA) revealed the presence of a distinct N protein from four Brazilian tospoviruses: *Tomato spotted wilt virus* (TSWV) [16], *Tomato chlorotic spot virus* (TCSV) [16], *Groundnut ringspot virus* (GRSV) [16] and *Zucchini lethal chlorosis virus* (ZLCV) [17] (Figure 1). A negligible cross-reaction was observed between BeNMV and TSWV and GRSV, strengthening the idea that BeNMV is a new Brazilian tospovirus. Usually, when polyclonal antibodies against the N proteins are utilized, an expressive cross-reaction is visualized between phylogenetically close species [18], [19], challenging the serological diagnosis.

### BeNMV has a very limited host range by mechanical inoculation

**Figure 2:**
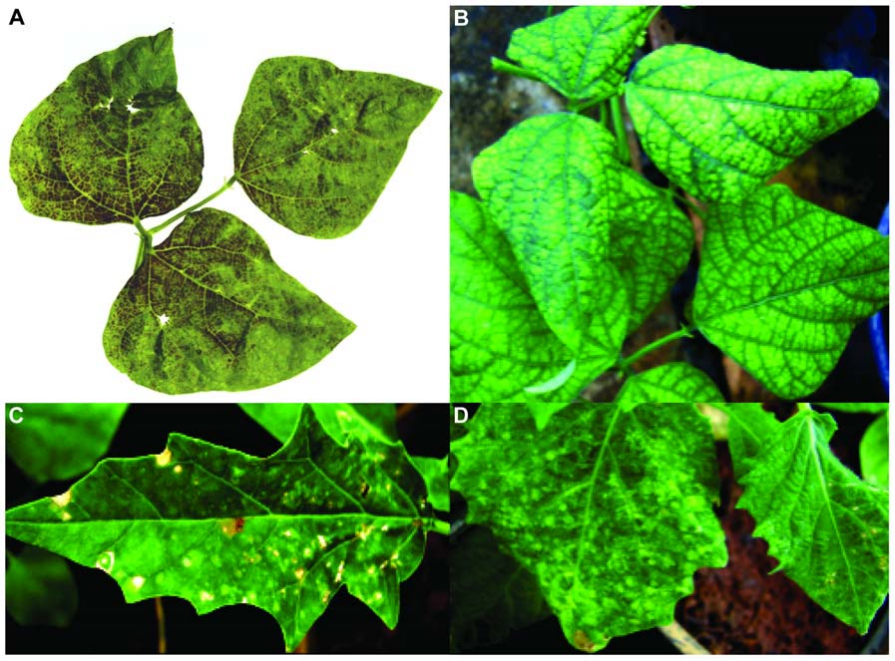
Field and greenhouse BeNMV-caused symptoms. A) *Phaseolus vulgaris* showing mosaic necrotic in the field. B), C) and D) *P. vulgaris* cv "Santana";, *Datura stramonium*, and *Physalis pubescens* presenting systemic symptoms in greenhouse 4 days post-inoculation, respectively.

The BeNMV isolate was inoculated to 20 distinct plants, including test-plants and some fabaceous plants to assess transmission capacity by mechanical inoculation, host range and symptomatology. Interestingly, only *Phaseolus vulgaris* cv. Santana, *Datura stramonium* L. and *P. pubescens* exhibited systemic symptoms post-inoculation (Table 1 and Figure 2). Specifically, foliar deformation, interveinal chlorosis and stunting were seen in *P. vulgaris* (Figure 2B). In *D. stramonium* the symptoms consisted of mottling, necrotic lesions, foliar deformation and stunting (Figure 2C), while *P. pubescens* plants exhibited mottling and stunting (Figure 2D). To further confirm that BeNMV did not replicate in other *P. vulgaris* varieties, a DOT-ELISA for nucleoprotein detection was performed to evaluate systemic infection, using both inoculated and upper leaves. Only *P. vulgaris* cv. Santana was found positive (Figure S1). Seven out of twenty tested plants reacted with local symptoms, which later did not evolve to systemic infection (Table 1). Crucially, this limited host range pattern is not observed in any other tospovirus species.

**Table 1:** Host range of BeNMV determined via mechanical inoculation 4 days post-inoculation.

Plant host	Local symptoms	Systemic symptoms
**Chenopodiaceae**		
*Chenopodium amaranticolor*	NL	-
*Chenopodium quinoa*	NL	-
*Chenopodium murale*	NL	-
**Cucurbitaceae**		
*Cucurbita pepo*	CS	-
**Fabaceae**		
*Phaseolus vulgaris* "Manteiga";	-	-
*Phaseolus vulgaris* "Santana";	-	St, IC, FD
*Phaseolus vulgaris* "BT2";	-	-
*Vigna angularis*	-	-
*Vigna unguiculata*	-	-
**Solanaceae**		
*Capsicum annuum*	-	-
*Capsicum chinense*	-	-
*Datura metel*	NL	-
*Datura stramonium*	-	NL, Mo, FD, St
*Solanum lycopersicum*	-	-
*Nicandra physaloides*	-	-
*Nicotiana benthamiana*	-	-
*Nicotiana rustica*	-	-
*Nicotiana tabacum* Samsun	NL, VC	-
*Nicotiana tabacum* TNN	NL	-
*Physalis pubescens*	CS	Mo, St

*CS* chlorotic spots; *FD* foliar deformation; *IC* interveinal chlorosis; *Mo* mottling; *NL* necrotic lesion; *St* Stunting; *VC* vein chloros.

The transmission by mechanical inoculation of BeNMV proved difficult, noticeable from its narrow host range. Despite the transmission to *P. vulgaris*, its natural host, the field-observed symptoms (Figure 2A) were not totally reproducible in greenhouse and, just one of three common bean varieties, was susceptible after many attempts. However, no transmission of a tospovirus to its natural host by mechanical inoculation had been reported for Alstroemeria necrotic streak virus (ANSV) [19]. For BeNMV, a more efficient transmission could be performed by one or more known thrips species. Alternatively, like ZLCV that has a peculiar host range and exclusive vector [17], [20], BeNMV could also have a new thrips species as vector, which would be different from those shared by other tospoviruses present in Brazil.

The difference between field symptoms and those observed under greenhouse conditions may have originated from genotypic differences between *P. vulgaris* collected in the field (cv. unknown) and the cultivar "Santana";. On the other hand, environmental interferences cannot be discarded as having played a part in the difference between symptoms. Furthermore, the allocated period for observation may not have been sufficient for the development of necrotic lesions.

### The S and M RNA of BeNMV has the same genetic organization as other tospoviruses

**Figure 3:**
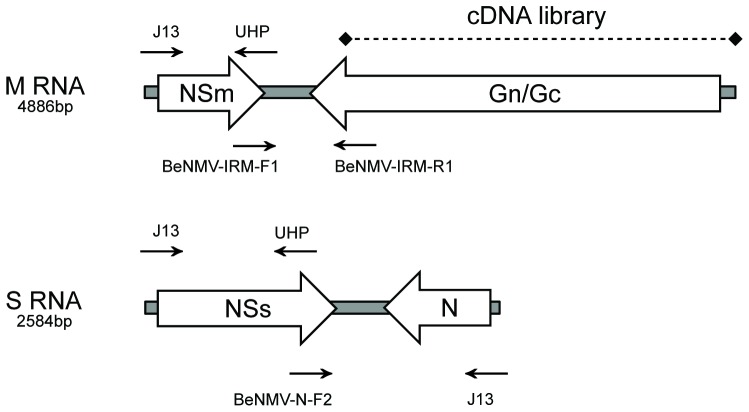
Cloning strategies for S and M RNA sequencing. The arrows indicate the primers' annealing positions. The dotted line shows the sequence obtained by cDNA library methodology.

The S and M RNA sequences of BeNMV were obtained from a cDNA library and by using degenerate and specific primers in reverse transcription coupled with polymerase chain reaction (RT-PCR) (Figure 3); in both cases cloning and automatic sequencing followed. The S RNA of BeNMV had 2,584 nucleotides (nt) and two ORFs corresponding to the N and NSs protein genes as in other tospoviruses. In the viral strand sense, the N protein ORF started at nucleotide position 2,508 and terminated at nucleotide 1,696, resulting in an ORF of 813 nt. Its encoded protein had 270 amino acids (aa) and a predicted molecular mass of 29.8 kDa. The NSs protein gene started at nucleotide position 61 and terminated at nucleotide 1,380, generating an ORF of 1,320 nt. Its encoded protein had 439 aa and a predicted molecular mass of 49.2 kDa. The S RNA presented 5′UTR and 3′UTR with 60 and 76 nt, respectively. The two ORFs were separated by an A/U-rich (79.4%) intergenic region (IR) of 315 nt, the second smallest among the tospoviruses' whose sequence is available (Table S1). The nucleotide and amino acid sequences were deposited in GenBank database under the accession number JN587268.

The M RNA had 4,886 nt and presented two ORFs corresponding to the Gn/Gc glycoprotein precursor and NSm protein genes as other tospoviruses. In the viral strand sense, GPp gene started at nucleotide position 4,803 and terminated at nucleotide 1,318, generating an ORF of 3,486 nt and 1,161 aa with a molecular mass prediction of 130.7 kDa. The NSm protein gene started at nucleotide position 65 and terminated at nucleotide 1,018, resulting in an ORF of 954 nt. Its encoded protein had 317 aa and a predicted molecular mass of 35.4 kDa. This NSm protein was the longest among all tospoviruses with M RNA sequenced (Table S1). The M RNA's A/U-rich (82.6%) IR presented 299 nt and the 5′UTR and 3′UTR with 64 nt and 83 nt, respectively. The nucleotide and amino acid sequences were deposited in GenBank database under accession number JN587269.

As observed in other tospoviruses [2], the S and M genomic segments of BeNMV presented an ambisense configuration. Both molecules showed higher proximity in their characteristics with SVNaV [14]. Structural characteristics such as length of the segments, open reading frames, and non-coding sequences, as well as predicted molecular mass of their proteins are similar (Table S1). For all S and M RNA encoded proteins, BeNMV showed more identity with SVNaV (Table S2).

In the topologic characterization of Gn/Cc glycoproteins, six putative N-glycosylation sites were predicted (Asn_123_, Asn_207_, Asn_320_, Asn_360_, Asn_521_, and Asn_1048_), but no O-glycosylation site was found. Similarly to other tospoviral GPp, two cleavage sites were found. The first was predicted in the N-terminal region between Leu_20_ and Asp_21_, after a putative signal peptide. The second site was found between Ala_468_ and Met_469_, potentially responsible for Gn and Gc processing. In general, three transmembrane domains were predicted (297 aa to 319 aa, 326 aa to 348 aa, and 1075 aa to 1097 aa), but using an old version of the TMHMM program, an additional two domains were indicated (6 aa to 24 aa and 442 aa to 460 aa). In summary, the glycoprotein precursor of BeNMV presented features observed in other tospoviruses [21] and, therefore, functions related to particle assemble and tospovirus-thrips interactions performed by the glycoproteins could be extrapolated for this new species [22]–[24].

Concerning the L RNA sequence, we previously demonstrated [15] that this genome segment of BeNMV presents similar genomic organization to the RNA-dependent-RNA polymerase genes of other tospovirus species characterized so far. However, BeNMV L protein is unique, it has 2,932 aa and a molecular mass of 335.9 kDa, being the largest RdRp for this genus at the present date.

### Phylogenetic analysis confirmed that BeNMV is, indeed, a distinct tospovirus species

The nucleoprotein (N) is commonly used for taxonomic classification [25]–[27] and new species should exhibit no more than 89% amino acid sequence identity to another member of the genus [2]. Therefore, to understand the evolutionary relationship between BeNMV and other tospoviruses, a data set composed of nucleoprotein sequences from distinct tospoviruses was collected. Notably, the N protein pairwise comparison showed that BeNMV differs from other tospoviruses from 17.2% to 52.2% (Table S2). This degree of divergence is considerable higher than the established threshold for new species acceptance (10%), confirming that BeNMV is a new tospovirus. Although we did not test all known tospoviruses, the lack of serological cross-reactivity in DOT-ELISA experiments (Figure 1) also supports this idea.

**Figure 4:**
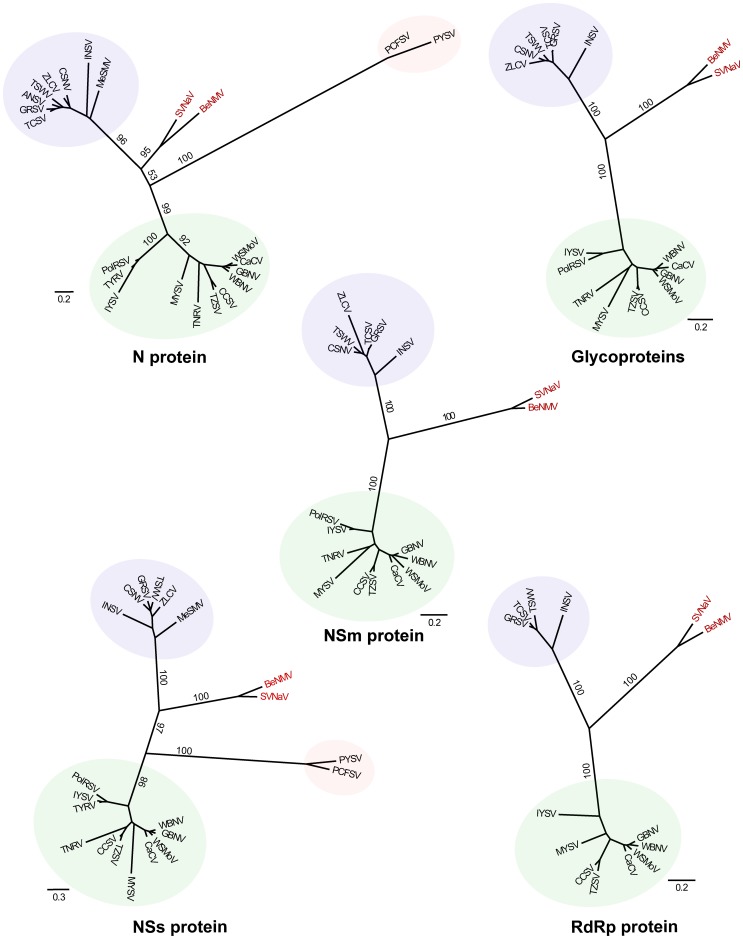
Phylogenetic relationships of *Tospovirus* species inferred using S and M RNA-encoded proteins. The trees were inferred with the maximum likelihood criterion implemented in the program RAxML. Node support was evaluated using non-parametric bootstrap resampling (500 replicates) and values are shown for key nodes. The BeNMV-SVNaV clade is shown in red. The shaded areas in purple, green and red represent the "Eurasian";, "American"; and PCFSV-PYSV clades, respectively. Tree inferred using **N protein** alignment: 23 taxa and 217 amino acids. Tree inferred using the **NSm protein** alignment: 18 taxa and 291 amino acids. Tree inferred using the **NSs protein** alignment: 21 taxa and 391 amino acids. Tree inferred using the **Glycoprotein** alignment: 18 taxa and 1007 amino acids. Tree inferred using the **RdRp protein** alignment: 14 taxa and 2811 amino acids. The complete viral names are found in the Table S2.

**Figure 5:**
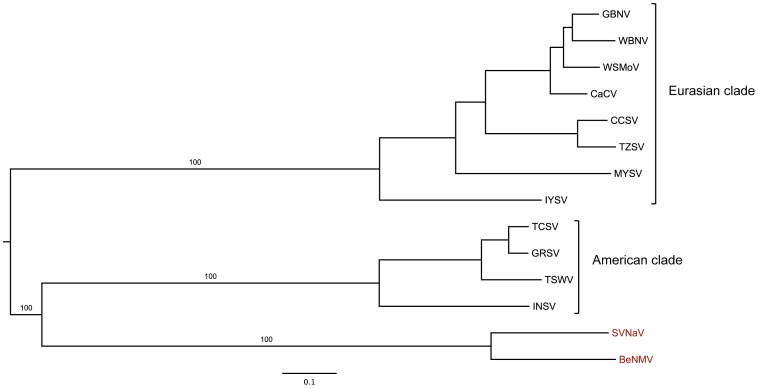
Phylogenetic relationships of *Tospovirus* species inferred using a concatenated dataset (N, NSs, NSm, Glycoprotein and RdRp). The tree was inferred with the maximum likelihood criterion implemented in the program RAxML. Node support was evaluated using non-parametric bootstrap resampling (500 replicates) and values are shown for key nodes. The tree is mid-point rooted for purposes of clarity. The BeNMV-SVNaV clade is shown in red and the previously described lineages are highlighted. The data set includes 14 taxa and 4721 amino acids.

The maximum likelihood tree based on the N protein is shown in Figure 4. As previously observed [13], [15], the American and Eurasian clades formed distinct monophyletic groups (bootstrap values of 98 and 98, respectively), and the Peanut chlorotic fan-spot virus (PCFSV) [28] and *Groundnut yellow spot virus* (PYSV) [29] (divergent viruses isolated from peanut) formed a monophyletic basal clade among tospoviruses. Crucially, the BeNMV was related to SVNaV, forming a well-supported monophyletic clade (bootstrap value of 93%), which was consistent with their pairwise distances (Table S2). The phylogenetic trees estimated from Nss, Nsm and Glycoprotein data sets are shown in Figure 4. Similarly, BeNMV consistently clustered with SVNaV, suggesting that reassortments were not involved in the origin of this BeNMV isolate. Furthermore, a phylogenetic tree inferred from the concatenated protein sequences (RdRp, N, NSs, NSm and Glycoprotein) robustly supports the observed clades (Figure 5), except for the PCFSV-PYSV clade, which was not included in this analysis.

These results confirmed that BeNMV is particularly distinct from other characterized tospovirus species, as previously suggested using only the RNA-dependent RNA polymerase (RdRp) protein [15]. The correspondence between N and RdRp protein phylogeny suggests that both genes can be used to understand the phylogenetic relationships among tospoviruses, with the advantage of using a more conserved region (RdRp) and, consequently, simplifying the PCR-based strategies for detection of highly divergent new viruses.

### BeNMV-SVNaV clade is a novel evolutionary lineage within the genus *Tospovirus*

The phylogenetic analysis (Figure 4 and Figure 5) also showed that the BeNMV-SVNaV clade was almost equidistant between the American and Eurasian lineages, suggesting that this clade constitutes a novel evolutionary lineage within tospoviruses. Importantly, when the PCFSV-PYSV clade was included in the analysis (N and NSs protein phylogenies) the BeNMV-SVNaV clade was more closely related to the American lineage (Figure 4). To further confirm this finding, the likelihood of the N protein best tree (lnL=−12018.58) (Figure 4) was compared to those estimated from alternative trees, constraining the BeNMV-SVNaV clade to be related to the Eurasian lineage (lnL=−12025.58) or to PCFSV-PYSV clade (lnL=−12024.78). Each of these alternative phylogenies was rejected with a Bayes factor above 5, further corroborating the suggested shared ancestry between the BeNMV-SVNaV clade and the American lineage. Altogether, these results support the notion that BeNMV-SVNaV clade is, indeed, the fourth clade of the genus *Tospovirus*.

This finding could have implications for tospovirus diagnosis and crop production. First, the available diagnostic reagents for tospoviruses, such as polyclonal serum and degenerated primers, might not detect viruses belonging to this new clade. Actually, several previously described primers designed for N gene amplification were not capable to detect BeNMV (data not shown), including those described for SVNaV [14]. Second, based on the diversity of the American and Eurasian clades, more species related to BeNMV-SVNaV clade probably remain to be discovery, increasing their economic impact on agriculture.

### Evidence of episodic diversifying selection in the branches leading to BeNMV-SVNaV clade and the American clade

**Figure 6:**
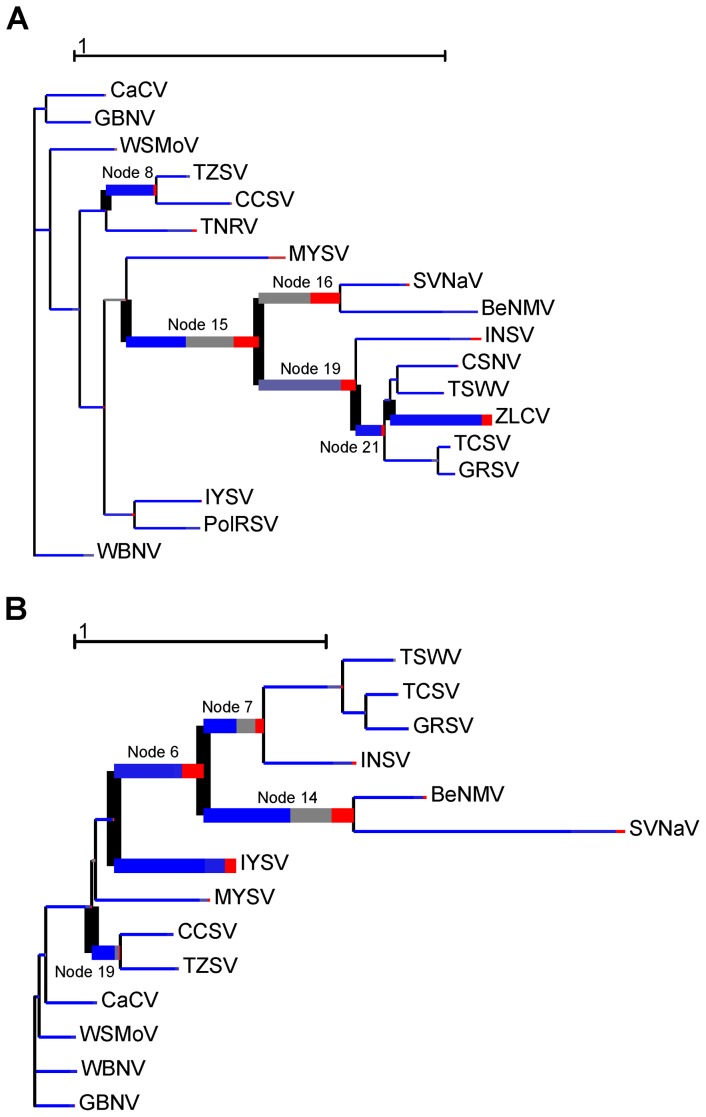
Episodic diversifying selection detected on NSm and RdRp alignments. Each tree is scaled on the expected number of substitutions/nucleotide. The hue of each color indicates strength of selection, with red corresponding to dn/ds >5, blue to dn/ds=0 and grey to dn/ds=1. The width of each color represents the proportion of sites in the corresponding class. Thicker branches have been classified as undergoing episodic diversifying selection (p>0.05).

Intriguingly, both BeNMV and SVNaV were isolated from common bean and soybean, respectively, which may indicate that viruses of this clade preferentially infect plants of the Fabaceae family, similar to the PCFSV-PYSV clade [28], [29]. In fact, if the different viral proteins were evolving in response to plant or insect host specificity, an increase in the ratio of non-synonymous (Ka) to synonymous substitutions (Ks) would be expected on those nodes leading to the different lineages. Therefore, a random effects branch-site model [30] was implemented in order to detect lineages on which a proportion of sites has evolved under positive selection. We found evidence of episodic diversifying selection only in the Nsm and RdRp datasets, along the branches leading to (*i*) the BeNMV-SVNaV and the American clade, to (*ii*) the BeNMV-SVNaV clade and to (*iii*) the American clade (Figure 6). It is hard to determine the biological constraints responsible for these events. Particularly, the occurrence of diversifying selection does not seem to be correlated with the width of plant host range. However, when we analysed viral vector host range it was possible to observe that the American clade is transmitted by several species of *Frankliniella* genus (at least 7), while those viruses from the Eurasian clade are predominantly transmitted by only two species of *Thrips* genus [13]. It is important to stress that sequence divergence is expected to influence the power of the branch-site tests because many synonymous sites might be saturated [31]. However, our results are reinforced by experimental data showing that both NSm and RdRp proteins are known to interact with the plant and insect restriction factors [32], [33], suggesting a classic evolutionary arms race. Actually, one of these studies showed that positive selection on the NSm protein was implicated in the tomato Sw-5 gene resistance breaking by TSWV [32].

## Materials and Methods

### Virus isolates, Host range and Symptomatology

The BeNMV isolate was isolated in São Paulo State in Brazil [15] and maintained in *P. pubescens* by mechanical inoculation [34]. The other tospovirus isolates (TSWV, TCSV, GRSV and ZLCV) were maintained in *D. stramonium*. In order to determine host range and symptoms, several plant species (Table 1) were mechanically inoculated with BeNMV. The plants were maintained in greenhouse and the onset of symptoms was observed up to four weeks post-inoculation.

### Virus purification and serology

Three weeks post mechanical inoculation, ribonucleocapsids were purified from 100 g of infected *P. pubescens* leaves following the protocol described by De Àvila et al. [34]. The purified ribonucleocapsids were injected in rabbits for polyclonal antibody production against N protein as described [34]. The serological differentiation was performed through DOT-ELISA between BeNMV and four tospovirus species found in Brazil (TSWV, TCSV, GRSV, and ZLCV) [16], [17]. The antisera to other tospoviruses were kindly supplied by Embrapa Vegetables (Brazil).

### RNA extraction and sequencing

Genomic RNA was extracted from BeNMV's ribonucleocapsids. For each 250 µL of sample were utilized 750 µL of Trizol LS (Invitrogen) following the manufacturer's recommendations. To determine the S and M RNA sequences two procedures were adopted. The first was the construction of a cDNA library using Universal Riboclone cDNA Synthesis System Kit (Promega). By this method, only the BeNMV's RNA-dependent RNA polymerase [15] and a truncated version of the glycoprotein precursor nucleotide sequences were obtained. Then, RT-PCR was performed to clone the remaining parts of the genome. Initially, J13 and UHP primers were utilized as described [25], where the latter was used for first-strand cDNA synthesis. Specific primers were then designed to complete the S and M RNA nucleotide sequences. The primer BeNMV-N-F2 (5′CTTCTGATGACAAGCTGCAAGGTA3′) and J13 were used to amplify the end of NSs open reading frame (ORF) and the remainder of S RNA. The primers BeNMV-IRM-F1 (5′GGCTGCAATAGATGAAGAGAATGAA3′) and BeNMV-IRM-R1 (5′GCCCTTTTGATTCTGTTATGACTTG3′) were used to amplify the end of Gn/Gc glycoprotein precursor ORF and M RNA intergenic region. The Figure 3 illustrates the cloning strategies for S and M RNA sequencing. M-MLV Reverse Transcriptase (Promega) was used for RT reactions and Platinum Taq DNA Polymerase (Invitrogen) was used for PCR. All procedures followed the manufacturer's instructions. All cDNA fragments were cloned in pGEM-T easy (Promega) and sequenced by chain-termination method using an automatic sequencer by Macrogen Corporation, Seoul, Korea. Sequence data were edited and assembled with Staden Package program [35].

### *In silico* analysis

N-glycosylation, O-glycosylation, and cleavage sites predictions were performed by NetNGlyc 1.0 Server, NetOGlyc 3.1 Server [36], and SignalP 3.0 [37], respectively. For transmembrane domain prediction was utilized the TMHMM Server 2.0 program.

### Evolutionary Analyses

All available tospovirus genome sequences were downloaded from GenBank: SVNaV (HQ728387, HQ728386), ANSV (GQ478668), CSNV (AF067068, AB600873, AF213675, AB274026), GRSV (AF513219, AY574055, AF513220), INSV (NC_003624, NC_003616), MeSMV (EU275149), TCSV (AF282982, AY574054, AF213674), TSWV (NC_002051, NC_002050), ZLCV (AF067069, AB274027, AF213676), CaCV (DQ256133, DQ256125), CCSV (AY867502, FJ822961), GBNV (NC_003619, NC_003620), IYSV (AF001387, AF214014), MYSV (NC_008300, NC_008307), PolRSV (EF445397, EU271753), TNRV (FJ489600, FJ947152), TYRV (AY686718), TZSV (NC_010489, NC_010490), WBNV (GU584184, GU584185), WSMoV (NC_003843, NC_003841), PCFSV (AF080526), and PYSV (AF013994). The complete viral names are available in Table S2.

The RNA coding sequences were aligned based on its corresponding amino acid translation using the software Muscle [38] implemented in TranslatorX web server [39]. The resulting alignments were inspected by eye and manually edited using Se-Al (v2.0a11 Carbon, http://tree.bio.ed.ac.uk/software/seal/), and all gap-containing sites were excluded. The level of substitution saturation was checked using Xia et al. (2003) [40] method implemented in DAMBE [41]. The third codon position of all alignments were saturated or nearly saturated (data not shown), therefore, phylogenetic trees were based on protein sequences alignments. The resulting data sets (available upon request) of 23 taxa for the N protein (217 amino acids, aa), 21 taxa for the NSs protein (391 aa), 18 taxa for the NSm protein (291 aa), 18 taxa for the Glycoproteins (1007 aa), 14 taxa for the RdRp protein (2811 aa). We also constructed a concatenated alignment of all proteins (N, NSs, NSm, Glycoproteins and RdRp) with 14 taxa (4721 aa). The phylogenetic relationships among the tospoviruses were inferred using maximum likelihood (ML) criterion implemented in RAxML [42], using the RAxML BlackBox web-server at CIPRES [http://www.phylo.org] [43]. The most appropriate model of protein evolution was selected with the software ProTest [44]. Node support was determined using non-parametric bootstrap resampling (500 replicates). The marginal likelihood of alternative topologies estimated using MrBayes 3.2 [45] were compared using Bayes factor. A log difference in the range of 3–5 is typically considered strong evidence in favor of the better model, while a log difference above 5 is considered very strong evidence [46]

The selection analyses were performed using the random effects branch-site model [47] available in www.datamonkey.org [48]. This method can identify branches in a tree with evidence of episodic diversifying selection. Therefore, each nucleotide alignment (N, NSs, NSm, Glycoproteins and RdRp) was submitted to the Datamonkey webserver.

## Supporting Information

Figure S1 **DOT-ELISA for BeNMV nucleoprotein detection in** ***Phaseolus vulgaris*** **varieties.** Polyclonal antibody against BeNMV nucleoprotein was used. A. Extract of inoculated leaves. B. Extract of upper leaves (not mechanically inoculated). Uninfected *P. vulgaris* was used as negative control (C−). Infected *Physalis pubescens* was used as positive control (C+). The dots are 1∶100 dilutions (leaf mass per volume of 0.5× PBS (g/ml).
(DOCX)

Table S1 **Characteristics of the S and M RNA for the avaiable tospoviruses.**
(DOCX)

Table S2 **Sequence identity comparison (%) of BeNMV proteins from S and M RNA.**
(DOCX)
