# Supplementary material for: Characterization of Bean Necrotic Mosaic Virus: A Member of a Novel Evolutionary Lineage within the Genus Tospovirus
Source: PLoS One. 2012 Jun 8;7(6):e38634. doi: 10.1371/journal.pone.0038634 (PMC3371012; doi:10.1371/journal.pone.0038634)
Supplement: Table S1 — Characteristics of the S and M RNA for the avaiable tospoviruses. (DOCX) [file pone.0038634.s002.docx]

| Virus | S RNA  length (nt) | 5'UTR (nt) | 3'UTR (nt) | Intergenic  region (nt) | N protein (aa) | N protein  mass (kDa) | NSs protein (aa) | NSs protein  mass (kDa) |
| --- | --- | --- | --- | --- | --- | --- | --- | --- |
| ANSV | - | - | - | - | 258 | 28.7 | - | - |
| **BeNMV** | 2,584 | 60 | 76 | 315 | 270 | 29.8 | 439 | 49.2 |
| CaCV | 3,477 | 66 | 67 | 1,196 | 275 | 30.6 | 439 | 49.6 |
| CCSV | 3,172 | 66 | 64 | 825 | 277 | 30.4 | 460 | 51.8 |
| CSNV | 2,940 | 79 | 152 | 531 | 260 | 29.2 | 464 | 51.7 |
| GBNV | 3,057 | 66 | 67 | 773 | 276 | 30.6 | 439 | 49.5 |
| GRSV | - | - | - | - | 258 | 28.7 | - | - |
| INSV | 2,992 | 62 | 149 | 642 | 262 | 28.7 | 449 | 51.1 |
| IYSV | 3,105 | 70 | 70 | 811 | 273 | 30.3 | 443 | 50 |
| MeSMV | 3,283 | 80 | 159 | 887 | 262 | 29.5 | 455 | 51 |
| MYSV | 3,232 | 68 | 67 | 847 | 279 | 31 | 469 | 53.1 |
| PCFSV | 2,833 | 67 | 79 | 455 | 270 | 31 | 472 | 52.3 |
| PolRSV | 2,484 | 72 | 72 | 183 | 274 | 30.1 | 443 | 50.1 |
| PYSV | 2,970 | 57 | 76 | 653 | 246 | 28.1 | 480 | 53.4 |
| SVNaV | 2,603 | 58 | 70 | 318 | 277 | 30.7 | 440 | 49.8 |
| TCSV | - | - | - | - | 258 | 28.5 | - | - |
| TNRV | 3,023 | 65 | 66 | 690 | 281 | 30.9 | 451 | 51.3 |
| TSWV | 2,916 | 88 | 153 | 503 | 258 | 28.8 | 464 | 52.4 |
| TYRV | 3,061 | 71 | 71 | 762 | 274 | 29.9 | 443 | 50.2 |
| TZSV | 3,279 | 64 | 64 | 934 | 278 | 30.6 | 459 | 51.9 |
| WBNV | 3,401 | 66 | 67 | 1,120 | 275 | 30.5 | 439 | 49.6 |
| WSMoV | 3,534 | 66 | 65 | 1,255 | 275 | 30.6 | 439 | 49.7 |
| ZLCV | - | - | - | - | 260 | 29 | - | - |
|  | M RNA length (nt) | 5'UTR (nt) | 3'UTR (nt) | Intergenic region (nt) | GPp (aa) | GPp mass (kDa) | NSm protein (aa) | NSm protein mass (kDa) |
| **BeNMV** | 4,886 | 64 | 83 | 299 | 1,161 | 130.7 | 317 | 35.4 |
| CaCV | 4,823 | 56 | 47 | 427 | 1,121 | 127.2 | 308 | 34.1 |
| CCSV | 4,704 | 54 | 45 | 303 | 1,123 | 127.5 | 309 | 34.6 |
| CSNV | - | - | - | - | 1,135 | 127.5 | 303 | 34 |
| GBNV | 4,801 | 56 | 47 | 408 | 1,121 | 127.3 | 307 | 34.2 |
| GRSV | - | - | - | - | 1,133 | 127.5 | 303 | 33.8 |
| INSV | 4,972 | 85 | 169 | 473 | 1,110 | 124.8 | 303 | 34.1 |
| IYSV | 4,838 | 63 | 49 | 379 | 1,136 | 128.5 | 311 | 34.7 |
| MYSV | 4,815 | 58 | 48 | 398 | 1,127 | 128.2 | 308 | 34.3 |
| PolRSV | 4,689 | 62 | 28 | 267 | 1,135 | 128.5 | 307 | 34.1 |
| SVNaV | 4,955 | 57 | 92 | 267 | 1,195 | 134.3 | 316 | 35.3 |
| TCSV | - | - | - | - | 1,134 | 127.7 | 303 | 33.9 |
| TNRV | 4,716 | 59 | 48 | 307 | 1,122 | 128 | 310 | 34.2 |
| TSWV | 4,821 | 100 | 84 | 320 | 1,135 | 127.3 | 302 | 33.7 |
| TZSV | 4,945 | 54 | 46 | 546 | 1,122 | 127.5 | 309 | 34.5 |
| WBNV | 4,794 | 55 | 47 | 402 | 1,121 | 127.1 | 307 | 34.3 |
| WSMoV | 4,880 | 55 | 47 | 473 | 1,121 | 127.6 | 312 | 35 |
| ZLCV | - | - | - | - | 1,135 | 127.5 | 303 | 34.1 |

Supplementary Table 1. Characteristics of the S and M RNA for the avaiable tospoviruses.
