# Supplementary material for: Characterization of Bean Necrotic Mosaic Virus: A Member of a Novel Evolutionary Lineage within the Genus Tospovirus
Source: PLoS One. 2012 Jun 8;7(6):e38634. doi: 10.1371/journal.pone.0038634 (PMC3371012; doi:10.1371/journal.pone.0038634)
Supplement: Table S2 — Sequence identity comparison (%) of BeNMV proteins from S and M RNA. (DOCX) [file pone.0038634.s003.docx]

**Supplementary Table 2.** Sequence identity comparison (%) of BeNMV proteins from S and M RNA

|  | N | NSs | GPp | NSm |
| --- | --- | --- | --- | --- |
| BeNMV | 100 | 100 | 100 | 100 |
| SVNaV | 52.2 | 61.6 | 64.5 | 75.2 |
| ANSV | 34.4 | - | - | - |
| CSNV | 35.5 | 18.8 | 36.0 | 37.6 |
| GRSV | 32.2 | - | 35.1 | 39.7 |
| INSV | 32.3 | 20.7 | 32.7 | 39.1 |
| MeSMV | 35.9 | 20.0 | - | - |
| TCSV | 34.1 | - | 35.3 | 38.9 |
| TSWV | 32.2 | 18.4 | 35.4 | 36.6 |
| ZLCV | 35.9 | - | 35.6 | 33.8 |
| CaCV | 27.1 | 18.2 | 31.5 | 36.5 |
| CCSV | 30.1 | 15.8 | 31.9 | 34.2 |
| GBNV | 28.5 | 17.6 | 32.2 | 36.8 |
| IYSV | 30.7 | 18.2 | 31.4 | 34.9 |
| MYSV | 28.5 | 16.2 | 30.6 | 34.3 |
| PolRSV | 31.6 | 18.7 | 30.3 | 35.7 |
| TNRV | 30.9 | 18.3 | 32.3 | 37.5 |
| TYRV | 30.5 | 18.9 | - | - |
| TZSV | 27.1 | 16.3 | 31.8 | 34.8 |
| WBNV | 27.9 | 17.3 | 31.6 | 36.2 |
| WSMoV | 28.2 | 18.0 | 31.9 | 36.3 |
| PCFSV | 18.0 | 14.9 | - | - |
| PYSV | 17.2 | 15.4 | - | - |

Acronyms: Bean necrotic mosaic virus (BeNMV), Soybean vein necrosis associated virus (SVNaV), Alstroemeria necrotic streak virus (ANSV), Chrysanthemum stem necrosis virus (CSNV), *Groundnut ringspot virus* (GRSV), *Impatiens necrotic spot virus* (INSV), Melon severe mosaic virus (MeSMV), *Tomato chlorotic spot virus* (TCSV), *Tomato spotted wilt virus* (TSWV), *Zucchini lethal chlorosis virus* (ZLCV), Capsicum chlorosis virus (CaCV), Calla lily chlorotic virus (CCSV), *Groundnut bud necrosis virus* (GBNV), Iris yellow spot virus (IYSV), Melon yellow spot virus (MYSV), Poligonum ringspot virus (PolRSV), Tomato necrotic ringspot virus (TNRV), Tomato yellow ring virus (TYRV), Tomato zonate spot virus (TZSV), Watermelon bud necrosis virus (WBNV), *Watermelon silver mottle virus* (WSMoV), Peanut chlorotic fan-spot virus (PCFSV), and *Peanut yellow spot virus* (PYSV). (-) Indicate the sequences are not available in GenBank database.
