# Supplementary material for: Characterization of Bean Necrotic Mosaic Virus: A Member of a Novel Evolutionary Lineage within the Genus Tospovirus
Source: PLoS One. 2012 Jun 8;7(6):e38634. doi: 10.1371/journal.pone.0038634 (PMC3371012; doi:10.1371/journal.pone.0038634)
Supplement: Supplementary file 3 [file pone.0038634.s001.docx]

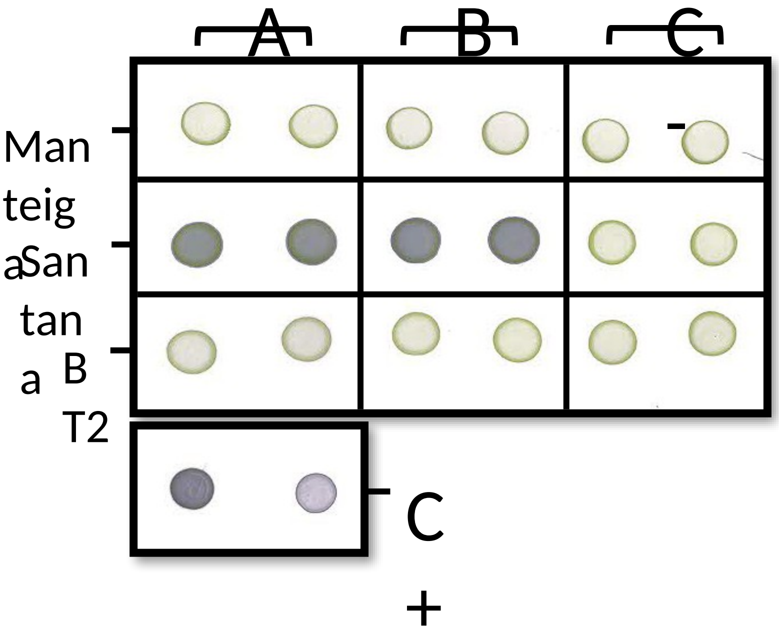
A
B
C
Manteiga
Santana
BT2
-
C
+
